# Progressive Medicine, Vol. I, 1901

**Published:** 1903-03

**Authors:** 


Progressive Medicine, Vol. I, 1901.—A quarterly digest of advances, discoveries and improvements in the medical and surgical sciences. Edited by Hobart Amory Hare, M. D., Professor of Therapeutics and Materia Medica in the Jefferson Medical College of Philadelphia. Octavo, handsomely bound in cloth, 430 pages, 11 illustrations. Per annum, in cloth-bound volumes, $10.00. Lea Brothers & Co., Philadelphia and New York.

This volume is replete with valuable matter. Those who have kept up with these volumes appreciate them, for they bring up to date all that is going on in the science of medicine. T. J. B.

